# Supplementary material for: Integrated analysis of the aqueous humor microbiome and lens capsule transcriptome in high myopia cataract: a pilot study
Source: Front Med (Lausanne). 2026 Jun 16;13:1845205. doi: 10.3389/fmed.2026.1845205 (PMC13314463; doi:10.3389/fmed.2026.1845205)
Supplement: Supplementary file 3 [file Data_Sheet_1.zip › 4.Meta_Statistic/C372089/T_test/Order/diff_Order_top10_boxplot_each1.pdf]

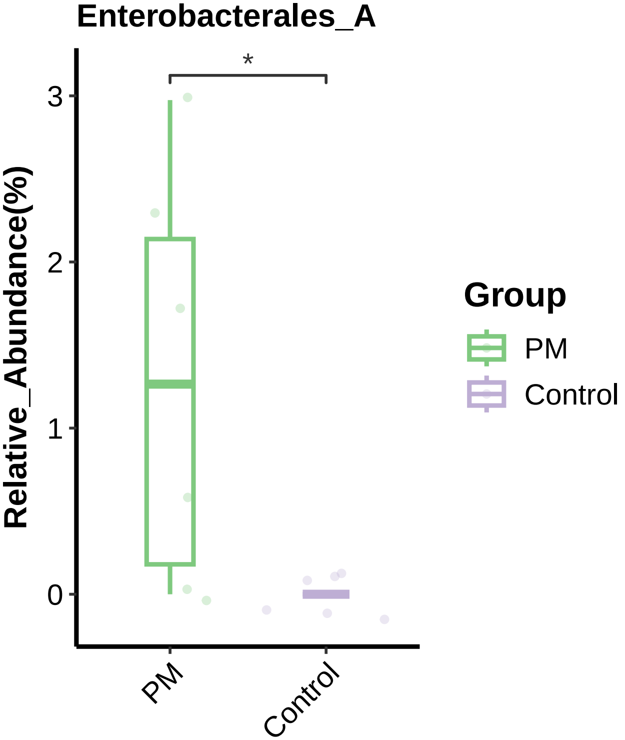

Enterobacterales_A
*
Relative_Abundance(%)
3
2
1
0
PM
Control
Group
PM
Control
